# Patient-Reported Favorable Outcomes of a Minimum Five-Year Follow-Up After Medial Patellofemoral Ligament (MPFL) Reconstruction: A Systematic Review and Meta-Analysis

**DOI:** 10.7759/cureus.80160

**Published:** 2025-03-06

**Authors:** Daniel I Razick, Muzammil Akhtar, Faith Sumandea, Tri Huynh, David Adams, Lara Ali, Adam A Razick, Jimmy Wen, Amir A Jamali

**Affiliations:** 1 Surgery, California Northstate University College of Medicine, Elk Grove, USA; 2 Family Medicine, California Northstate University College of Medicine, Elk Grove, USA; 3 Orthopaedic Surgery, California Northstate University College of Medicine, Elk Grove, USA; 4 Orthopaedic Surgery, Temple University, Philadelphia, USA; 5 Obstetrics and Gynaecology, California Northstate University College of Medicine, Elk Grove, USA; 6 Psychology, University of California Los Angeles, Los Angeles, USA; 7 Physical Medicine and Rehabilitation, California Northstate University College of Medicine, Elk Grove, USA; 8 Orthopedic Surgery, Joint Preservation Institute, Sacramento, USA

**Keywords:** medial patellofemoral reconstruction, mpfl, mpfl reconstruction, patella, patellofemoral instability

## Abstract

Medial patellofemoral ligament (MPFL) reconstruction is a procedure performed to address patellofemoral instability. The primary objective of this paper is to evaluate studies reporting the outcomes of MPFL reconstruction at a minimum five-year follow-up through analysis of patient-reported outcomes (PROs), re-dislocations, and range of motion. We hypothesize that MPFL reconstruction will demonstrate excellent PROs, low redislocation rates, and full range of motion. A search following guidelines established by the Preferred Reporting Items for Systematic Reviews and Meta-Analyses (PRISMA) was performed in three databases on March 23, 2024: PubMed, Embase, and the Cochrane Library. The query was performed utilizing the Boolean search phrase “(Medial patellofemoral ligament reconstruction OR MPFL reconstruction). There were no restrictions set to the search. Studies were included if they reported on outcomes of MPFL reconstruction at a minimum follow-up of five years. Twelve studies published between 2007 and 2023, containing 498 patients and 521 knees were included with a mean follow-up period of 98 months (60-215). Autologous gracilis grafts were the most common source used for MPFL reconstruction. Statistically significant improvements were found in preoperative to postoperative Kujala, Lysholm, and Tegner scores (p < 0.001). The overall re-dislocation rate across all studies was 5.75%, and the reoperation rate was 0.38%. MPFL reconstruction demonstrates excellent PROs with low redislocation rates at a minimum five-year follow-up and can be safely considered in patients with patellar instability.

## Introduction and background

Patellar injuries, such as patellar dislocation, are severe and happen most frequently among teenage patients, especially among athletes, and young adults [1]. Patellar instability and dislocation occur when the patella disarticulates from the patellofemoral joints [2]. It was found that, following patellar dislocation, medial patellofemoral ligament (MPFL) damage and tears occurred in most patients, presenting as pain, tenderness, and large hemarthrosis [1]. The MPFL is the primary ligament that stabilizes and constrains the lateral movement of the patella, keeping the patella centered in the trochlear groove [1]. The MPFL runs between the medial epicondyle of the femur and the adductor tubercle, superior to the origin of the medial collateral ligament [1]. Once damaged, it could cause loss of range of motion, lateral patellar instability, and incorrect patellar tracking [3,4].

The MPFL was first described in 1957 as a transverse reinforcement between the tendon of the medial head of the gastrocnemius and the base of the patella [5]. It was then discovered that the MPFL was the primary ligamentous restraint of the patella, providing 50-60% of restraining force preventing lateral dislocation [6]. To address MPFL damage, there are several surgical and non-surgical methods of repair. For non-surgical methods, immobilization of the knee and physical therapy are among some of the techniques employed [7]. First described in 1992, there are many different methods of surgical reconstruction. The original method of MPFL reconstruction utilizes polyester ligaments fixed at the medial condyle [8]. Currently, different graft sources have been used, including autografts and allografts of the gracilis, semitendinosus, and portions of the quadriceps [4,9]. Furthermore, there are different graft fixation techniques, such as bone tunnel fixation (using a screw in the femur) or soft-tissue fixation with sutures, or looping the graft through the adductor magnus tendon [10] with suture anchors. Generally, it is believed that operative MPFL reconstruction offers a reliable method of improving patellar pain and instability. Despite the wide range of available options in MPFL reconstruction, there is currently no consensus regarding the procedure that would yield the most favorable result [4].

The primary objective of this paper was to evaluate studies reporting the outcomes of MPFL reconstruction at a minimum five-year follow-up through analysis of patient-reported outcomes (PROs), dislocation/subluxation rates, and persistent joint instability. We hypothesized that MPFL reconstruction will demonstrate favorable medium to long-term outcomes.

## Review

Methods

Search Strategy

A search following guidelines established by the Preferred Reporting Items for Systematic Reviews and Meta-analyses (PRISMA) was performed in three databases on March 23, 2024: PubMed, Embase, and the Cochrane Library. The query was performed utilizing the Boolean search phrase “((Medial patellofemoral ligament reconstruction OR MPFL reconstruction) AND outcome).” There were no restrictions set to the search. Studies were included if they reported on outcomes of MPFL reconstruction at a minimum follow-up of 5 years. Exclusion criteria included case reports, review articles, conference abstracts, studies performed in animals, articles not in English, expert opinions, letters to editors, and studies in which outcomes pertaining to MPFL reconstruction were not specified. This study was registered on PROSPERO (ID: CRD42024534487).

Study Selection

Two independent reviewers (MA and DIR) reviewed studies for eligibility criteria from the initial database search. A third senior author was available for any disputes. When multiple studies from the same author were found, only the one with the longest follow-up period was included to not have the same patients from a single author counted multiple times. All included articles underwent rigorous reference search to determine whether additional studies could be added to the systematic review.

Data Extraction

Study variables extracted from each article included author, publication year, journal, level of evidence (LOE), study time period, study design, number of patients, sex, mean age, follow-up period, prior surgeries, preoperative patellar cartilage lesions, preoperative trochlear characteristics, Caton-Deschamps Index (CDI), complication rates, pre- and postoperative range of motion, pre- and postoperative radiographic measures, dislocation/subluxation rates, and patient-reported outcomes. All extracted data were compiled for analysis using Microsoft Word (Microsoft Office 2011; Microsoft, Redmond, WA).

Quality Assessment and Risk of Bias (ROB)

The methodological quality of studies was assessed using the methodological index for non-randomized studies (MINORS) checklist. The MINORS items are scored 0 (not reported), 1 (reported but inadequate), or 2 (reported and adequate), with a maximum possible score of 16 for non-comparative studies (from eight categories) and 24 for comparative studies (from 12 categories). Two authors scored each article in the systematic review. Each author scored the article individually before reviewing their scores, and any discrepancies were resolved by re-reviewing the articles until a unanimous consensus was met. The ROB was determined based on the overall MINORS score. The ROB was considered high if the MINORS score was 0-8 (0-16 for comparative studies), moderate if the MINORS score was 9-12 (16-20 for comparative studies), and low if the MINORS score was 13-16 (21-24 for comparative studies) [11].

Statistical Analysis

Descriptive statistics (mean, percentage, standard deviation, range, median) are reported in this review when applicable and when available. A meta-analysis consisting of preoperative and postoperative Kujala [12], Lysholm [13], and Tegner [14] scores was performed to compare outcomes and determine if there was a statistically significant mean difference. Forest plots depicting data from all studies, the overall significance, and the I2 statistic to assess heterogeneity were created using Cochrane’s Reviewer Manager web application (RevMan; Computer program, version 5.4; The Cochrane Collaboration, 2020). A p-value of less than 0.05 was considered statistically significant.

Results

Article Selection Process

**Figure 1:**
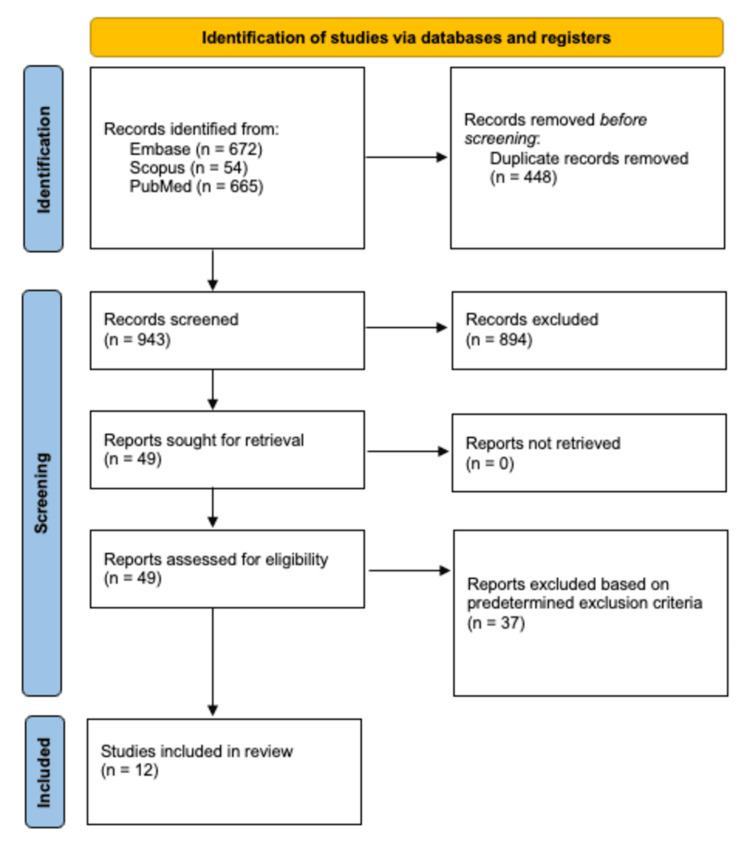
Preferred Reporting Items for Systematic Reviews and Meta-Analyses (PRISMA) flow diagram depicting the article selection process

Upon the initial search of the Scopus, Embase, and PubMed databases, 1,391 studies were identified, of which 448 duplicates were removed. The remaining 943 studies underwent full title and abstract review, of which 894 were removed based on our predetermined exclusion criteria. The remaining 49 studies underwent full-text review. A total of 37 of these studies were excluded as they did not fit our predetermined inclusion criteria, which included incorrect outcomes and insufficient follow-up periods. The remaining 12 were included in this systematic review. The PRISMA flow diagram depicting our search strategy and method of selecting articles is depicted in Figure 1.

Methodological Index and ROB Assessment

The mean ± standard deviation (range) of the MINORS score for comparative studies discussing mid- to long-term outcomes of MPFL reconstruction was 18.0 ± 2.0 (16-20). For noncomparative studies, the MINORS score was 11.6 ± 0.59 (11-12). Overall, the ROB was moderate for all 12 studies. The ROB assessment and total MINORS score are reported for each study in Table 1.

**Table 1:** Risk of bias assessment using the MINORS criteria MINORS: Methodological Index for Non-randomized Studies

	Criteria for non-comparative studies								Additional criteria for comparative studies				
Authors	A clearly stated aim	Inclusion of consecutive patients:	Prospective collection of data	Endpoints appropriate to the aim of the study	Unbiased assessment of the study endpoint: blind evaluation/ double-blind	Follow-up period appropriate to the aim of the study:	Loss to follow-up less than 5%	Prospective calculation of the study size:	An adequate control group:	Contemporary groups:	Baseline equivalence of groups	Adequate statistical analyses	Sum
Boelch et al. [15]	2	2	0	2	2	2	2	0					12
Gao et al. [16]	2	2	0	2	2	2	2	0					12
Gupta et al. [17]	2	2	0	2	2	2	1	0					11
Ji et al. [18]	2	2	0	2	2	2	2	0	2	1	1	0	16
Leite et al. [19]	2	2	0	2	2	2	1	0					11
Marcheggiani Muccioli et al. [20]	2	2	0	2	2	2	2	0					12
Nomura et al. [21]	2	2	0	2	2	2	2	0					12
Shatrov et al. [22]	2	2	0	2	2	2	1	0					11
Shimizu et al. [23]	2	2	0	2	2	2	1	0					11
Sillanpää et al. [6]	2	2	0	2	2	2	2	0					12
Xie et al. [24]	2	2	1	2	2	2	2	0	2	2	2	1	20
Zhang et al. [25]	2	2	1	2	2	2	1	0					12

Study Characteristics and Patient Demographic Information

The 12 studies [6,15-25] included in this review were published between 2007 and 2023. Ten studies had a retrospective design [6,15-23], and two had a prospective design [24,25]. The LOE was I in one study, III in four studies, and IV in seven studies. Across all 12 included studies, a total of 498 patients (521 knees; 34.0% male, 66.0% female) were identified with mean ages ranging from 14 to 25.4 years and mean follow-up times ranging from 5 to 12.4 years. Gracilis tendon autografts were the most common graft sources and isolated MPFL reconstructions were the most common procedure performed. Ji et al. [18] compared MPFL reconstruction using the modified semi-tunnel bone bridge fixation technique (Group A) versus the suture anchor fixation technique (Group B). Xie et al. [24] compared MPFL reconstruction with suture augmentation (Group A) versus without suture augmentation (Group B). Table 2 summarizes the author of each study, study period, LOE, study period, graft type/technique, number of patients/knees, sex, mean age, and mean follow-up time.

**Table 2:** Study characteristics and patient demographics In the study by Ji et al. [18], Group A was treated with modified semi-tunnel bone bridge fixation and Group B with suture anchor fixation. In the study by Xie et al. [24], Group A was treated with polyester suture augmentation and Group B without augmentation. Age and follow-up are reported as mean ± standard deviation (range) when possible. LOE, level of evidence; NR, not reported; TTA, distal tibial tuberosity; TTO, tibial tubercle osteotomy; M/F, male/female; MPFLR, medial patellofemoral ligament reconstruction

Authors	Study Period	LOE	Graft	Procedures	Patients/Knees (M/F)	Age (Years)	Follow-up (Months)
Boelch et al. [15]	2010-2012	4	Gracilis tendon autograft	Isolated MPFLR	50/50 (19/31)	20.9 (10-47)	69.8 (60-86)
Gao et al. [16]	2012-2013	4	Gracilis tendon autograft	Isolated MPFLR	66/80 (17/49)	21.3 ± 7.8 (13-50)	66.1 ± 5.5 (60-78)
Gupta et al. [17]	2012-2015	4	Hamstring tendon autograft	Isolated MPFLR	35/37 (29/6)	19.6 ± 8	81.6 ± 16.1
Ji et al. [18] (Group A)	2007-2010	3	Semitendinosus autograft	Isolated MPFLR	30/30 (12/18)	23.4 ± 3.6	86 (60-95)
Ji et al. [18] (Group B)					30/30 (10/20)	23.3 ± 3.8	
Leite et al. [19]	2011-2015	3	Gracilis tendon autograft	Isolated MPFLR	19/19	14 (11-17)	Minimum 60
Marcheggiani Muccioli et al. [20]	2012-2015	4	Fascia lata allograft	Isolated MPFLR (53%), MPFLR + TTA distalization (6%), MPFLR + TTA distalization/medialization (29%), MPFLR + TTA distalization/medialization + trochleoplasty (12%)	17/17 (11/6)	21.7 ± 5.1 (15.9-30.3)	64.3 ± 12.5
Nomura et al. [21]	1988-1997	4	Tape-type and mesh-type artificial ligament (Leeds-Keio ligament)	Isolated MPFLR (42%), MPFLR + lateral release (58%)	22/24	22/5 ± 10.6 (13-48)	142.8 ± 32.4 (102-206.4
Shatrov et al. [22]	2000-2011	4	Gracilis tendon autograft	Isolated MPFLR	54/54 (17/37)	25.4	148.8 ± 18 (120-177.6)
Shimizu et al. [23]	1999-2012	4	Semitendinosus autograft	MPFLR with Insall’s proximal realignment	15/20 (2/13)	19.9 (11-41)	123 (60-215)
Sillanpää et al. [6]	1994-2000	3	Adductor magnus tenodesis	Isolated MPFLR	15/15	20.2 (19-22)	121.2 (96-156)
Xie et al. [24] (Group A)	2004-2006	2	Semitendinosus autograft	MPFLR with unspecified number of patients undergoing concomitant TTO	42/42 (6/36)	NR	Minimum 60
Xie et al. [24] (Group B)					43/43 (7/36)		
Zhang et al. [25]	2005-2010	3	Semitendinosus autograft	Isolated MPFLR	60/60	21 (16-32)	96 (72-120)

Caton-Deschamp Index (CDI), Trochlear Dysplasia, Range of Motion, and Other Radiographic Measurements

The mean preoperative and postoperative CDI was reported in four studies (range: 1.1-1.4) and two studies (range: 1-1.2). The degree of preoperative trochlear dysplasia according to the Dejour classification system was reported in four studies. One or more postoperative radiographic measures were reported in seven studies, with the most common measures being the patellar tilt angle, lateral patellar angle, and lateral patellar translation. Preoperative and postoperative range of motion was reported in two (range: 121.4°-123°) and four studies (range: 130.8°-149°). Table 3 summarizes the CDI, degree of trochlear dysplasia, range of motion, and additional radiographic measures reported in the included studies.

**Table 3:** Pre- and postoperative ranges of motion (ROM), radiographic measures, Caton-Deschamp Index (CDI), preoperative cartilage lesions, and preoperative trochlear characteristics NR, not reported; TD, trochlear dysplasia; TT-TG, tibial tuberosity-trochlear groove

Authors	CDI			Preoperative Trochlear Dysplasia	Range of Motion			Other Radiographic Measurements				
	Preoperative		Postoperative		Preoperative	Postoperative		Measurement		Preoperative		Postoperative
Boelch et al. [15]	1.4		NR	No TD: 19.1%; TD Type A: 27.9%; TD Type B: 29.4%; TD Type C: 19.1%; TD Type D: 4.4%; Not classifiable: 4.4%	NR	149° (115°-165°)		NR				
Gao et al. [16]	NR			TD Type A: 51.3%; TD Type B: 25%; TD Type C: 15%; TD Type D: 8.7%	NR			Q angle	NR		14.5° ± 3.5° (5°-28°)	
Gupta et al. [17]	NR			TD patients excluded	NR			NR				
Ji et al. [18] (Group A)	NR			NR	NR			Patellar tilt angle	23.2° ± 2.3°		11.5° ± 1.6°	
								Lateral patellar angle	-7.4° ± 1.4°		5.1° ± 1.2°	
Ji et al. [18](Group A)	NR			NR	NR			Patellar tilt angle	23.7° ± 2.3°		14.9° ± 1.7°	
								Lateral patellar angle	-6.8° ± 1.5°		3.7° ± 1.1°	
Leite et al. [19]	1.3 ± 0.2	NR		No TD/TD Type A: 15.8%; Type B: 5.3%; Type C: 15.8%; Type D: 63.2%	NR			Patellar tilt angle	NR		23.3° ± 10.4°	
Marcheggiani Muccioli et al. [20]	1.3 ± 0.2	1.2 ± 0.2		NR	123° ± 34°	145° ± 6°		Patellar tilt angle	20.9° ± 5.2°		15.8° ± 8.5°	
Nomura et al. [21]	NR			NR	NR			Congruence angle	15.5° ± 17° (-8°-58°)		-9.7° ± 10.7° (-35°-10°)	
								Sulcus angle	148.8° ± 9° (138°-173°)		143.5° ± 10.6° (130°-170°)	
								Patellar height (Insall-Salvati Index)	1.1 ± 0.1 (0.8-1.4)		1.1 ± 0.1 (0.8-1.4)	
Shatrov et al. [22]	1.1 ± 0.1 (0.7-1.3)	1 ± 0.1 (0.7-1.2)		TD Type A: 74.1%; TD Type B: 14.8%; TD Type C: 11.1%	NR			TT-TG Distance	15.1 ± 2.9 (7-20) mm		NR	
Shimizu et al. [23]	NR			NR	NR			Sulcus angle	149.7° ± 11.2°		NR	
Sillanpää et al. [6]	NR			NR	NR	141° (130°-155°)		Sulcus angle	NR		145° (125°-166°)	
								Lateral patellar angle	NR		0° (-8°-10°)	
								Lateral patellar translation	NR		3 (0-7) mm	
Xie et al. [24](Group A)	NR			NR	NR			Patellar tilt angle	25.2° ± 16.6°		2° ± 7.3°	
								Lateral patellar translation	15.1 ± 5.6 mm		2.2 ± 5.1 mm	
								Lateral patellar angle	-17.7° ± 15.8°		5.7° ± 7.2°	
								Congruence angle	-27.8° ± 16.6°		3.3° ± 6.4°	
								TT-TG Distance	18.1 ± 3.9 mm		NR	
								Patella-trochlea Distance	-3.1 ± 2.4 mm		NR	
Xie et al. [24](Group B)	NR			NR	NR			Patellar tilt angle	27.8° ± 14.6°		8.9° ± 7.4°	
								Lateral patellar translation	14.7 ± 4.9 mm		6.4 ± 6 mm	
								Lateral patellar angle	-16.4° ± 14.9°		-6.7° ± 6.9°	
								Congruence angle	-24.4° ± 17.1°		-9.4° ± 6.6°	
								TT-TG Distance	19.4 ± 4.3 mm		NR	
								Patella-trochlea Distance	-3.2 ± 2.6 mm		NR	
Zhang et al. [25]	NR			NR	121.4° ± 12.7°		130.8° ± 5.8°	NR				

Patient-Reported Outcomes

The preoperative and postoperative Kujala score was reported in 10 (range: 51.1-68.9) and all studies (range: 80.3-97.8). The preoperative and postoperative Lysholm score was reported in six (range: 43.5-73.5) and six studies (range: 85.4-95.3). The preoperative and postoperative Tegner score was reported in seven (range: 2-6.2) and nine studies (range: 3-7.8). Table 4 summarizes the Kujala, Lysholm, and Tegner scores for all studies.

**Table 4:** Pre- and postoperative patient reported outcomes NR, not reported

Authors	Kujala			Lysholm					Tegner							
	Preoperative	Postoperative	P-value	Preoperative		Postoperative		P-value	Preoperative			Postoperative				P-value
Boelch et al. [15]	68.8 (19-92)	88.2 (49-100)	< 0.0001	71.3 (24-100)		88.4 (28-100)		< 0.0001	5.1 (2-9)			5.0 (2-9)				0.497
Gao et al. [16]	69.4 ± 7.9	96.1 ± 1.9	< 0.0001	73.5 ± 14.6		95.3 ± 3.4		< 0.0001	3.1 ± 1.3			5.9 ± 1.3				< 0.0001
Gupta et al. [17]	62.2 ± 19.0	90.3 ± 16.4	< 0.0001	68.2 ± 10.7		95.3 ± 8.0		< 0.0001	6.2 ± 2.1			5.8 ± 1.9				0.38
Ji et al. [18](Group A)	52.7 ± 3.7	89.9 + 3.7	NR	50.2 ± 3.7		90.7 ± 4.1		NR	NR							
Ji et al. [18](Group B)	51.1 ± 3.2	85.5 ± 5	NR	49.3 ± 3.4		86.4 ± 4.8		NR	NR							
Leite et al. [19]	52.1 ± 16.8	80.3 ± 17.3	< 0.001	NR					2 (0-4)	3 (2-4)			< 0.001			
Marcheggiani Muccioli et al. [20]	61.2 ± 18.1	82.1 ± 10.2	< 0.001	NR					3 (2-4)	5 (3-9)			< 0.001			
Nomura et al. [21]	63.2 ± 12.7 (26-85)	94.2 ± 7 (76-100)	< 0.0001	NR					NR							
Shatrov et al. [22]	NR	82.9 ± 15.3 (41-100)	-	NR					NR		4 ± 1.7 (1-9)			-		
Shimizu et al. [23]	65.5 ± 17	86.7 ± 14.9	< 0.05	NR					NR							
Sillanpää et al. [6]	NR	88 (57-100)	-	NR					NR		4 (2-8)				-	
Xie et al. [24](Group A)	68.9 ± 6.9	97.8 ± 6.4	< 0.001	52.4 ± 6.7	92.3 ± 3.1		< 0.001		3.3 ± 1.7		6.8 ± 1.3				< 0.001	
Xie et al. [24](Group B)	69.2 ± 7.4	88 ± 6.3	< 0.001	54.8 ± 7.5	85.4 ± 6.6		< 0.001		3.5 ± 1.8		5.5 ± 1.9				< 0.001	
Zhang et al. [25]	57.5 ± 8.6 (36-79)	88.9 ± 3.8 (73-95)	< 0.001	43.5 ± 10.2 (23-65)	89.7 ± 4.1 (80-96)		< 0.001		2.9 ± 0.8		7.8 ± 0.9				< 0.001	

**Figure 2:**
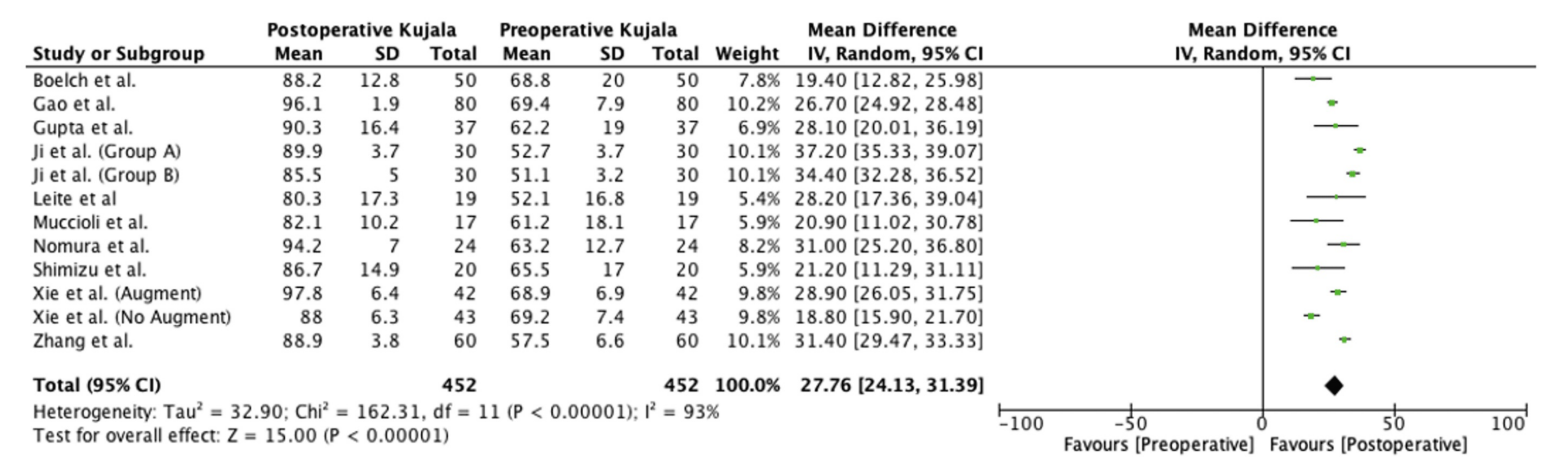
Preoperative versus postoperative Kujala scores SD, standard deviation; CI, confidence interval Source: Sillanpää et al. [6], Boelch et al. [15], Gao et al. [16], Gupta et al. [17], Ji et al. [18] (Group A), Ji et al. [18] (Group B), Leite et al. [19], Marcheggiani Muccioli et al. [20], Nomura et al. [21], Shatrov et al. [22], Shimizu et al. [23], Xie et al. [24] (Group A), Xie et al. [24] (Group B) and Zhang et al. [25]

**Figure 3:**
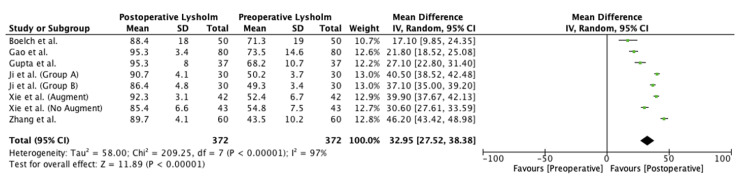
Preoperative versus postoperative Lysholm scores SD, standard deviation; CI, confidence interval Source: Boelch et al. [15], Gao et al. [16], Gupta et al. [17], Ji et al. [18] (Group A), Ji et al. [18] (Group B), Xie et al. [24] (Group A), Xie et al. [24] (Group B) and Zhang et al. [25]

**Figure 4:**
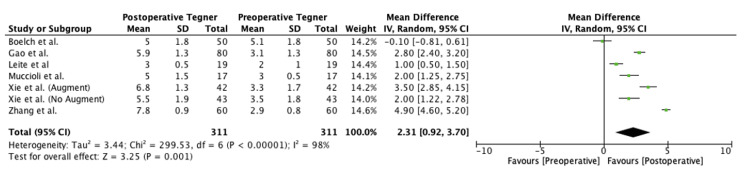
Preoperative versus postoperative Tegner scores SD, standard deviation; CI, confidence interval Boelch et al. [15], Gao et al. [16], Leite et al. [19], Marcheggiani Muccioli et al. [20], Xie et al. [24] (Group A), Xie et al. [24] (Group B) and Zhang et al. [25]

The meta-analysis in the present study found significant preoperative to postoperative improvement in the Kujala (mean difference (MD), 27.8 (95% confidence interval (CI), 24.1-31.4); p < 0.00001), Lysholm (MD, 33.0 (95% CI, 27.5-38.4); p < 0.00001), and Tegner scores (MD, 2.3 (95% CI, 0.9-3.7); p = 0.001). Forest plots for this meta-analysis are presented in Figures 2-4.

The preoperative and postoperative International Knee Documentation Committee (IKDC) score was reported in two (range: 47.8-58.6) [17,24] and three studies (range: 78.4-87.7) [17,22,24]. The two studies [17,24] reporting both preoperative and postoperative scores found significant preoperative to postoperative improvements.

Adverse Postoperative Events

Incidence of dislocations/subluxations was reported in all 12 studies, with an overall dislocation/subluxation rate of 5.75%. Gao et al. reported excellent postoperative satisfaction in 86% of patients [16]. Gupta et al. reported positive apprehension tests in 10.8% of knees postoperatively [17]. Marcheggiani Muccioli et al. reported anterior knee pain in 11.8% of patients [20]. Nomura et al. additionally provided Crosby/Insall and Kellgren/Lawrence grades with over 60% of patients showing excellent outcomes [21]. Shimizu et al. reported that 25% of patients presented postoperatively with a positive apprehension test, while one patient reported temporary joint stiffness, which resolved after manipulation under anesthesia (MUA) [23]. Xie et al. reported a failure rate defined by re-dislocation or patellar instability [24]. All patients in their study underwent MPFL reconstruction using semitendinosus tendons either with polyester suture augmentation or without polyester suture augmentation. Patients with suture augmentation had a failure rate of 2.4%, while those without augmentation had a failure rate of 23.39%. Table 5 summarizes postoperative dislocation/subluxation rates and additional outcomes.

**Table 5:** Dislocation/subluxation rates and additional outcomes NR, not reported

Authors	Dislocation/Subluxation Rates	Additional Outcomes
Boelch et al. [15]	Dislocation: 5.6%	NR
Gao et al. [16]	Dislocation: 3.0%	NR
Gupta et al. [17]	Dislocation: 0%	NR
Ji et al. [18] (Group A)	Dislocation: 6.7% (all underwent revision)	NR
Ji et al. [18] (Group B)	Dislocation: 13.3% (all underwent revision)	
Leite et al. [19]	Dislocation: 26.3%	NR
Marcheggiani Muccioli et al. [20]	Lateral dislocation: 5.8%	Anterior knee pain (11.8%)
Nomura et al. [21]	Lateral subluxation/dislocation: 8.3%	NR
Shatrov et al. [22]	Dislocation: 7.4% (all underwent revision)	Postoperative hematoma (1.9%), ACL reconstruction (1.9%), Arthroscopic arthrolysis of adhesions (1.9%)
Shimizu et al. [23]	Subluxation: 5.0%	Temporary joint stiffness (5%)
Sillanpää et al. [6]	Dislocation: 6.7% Subluxation: 13.3%	Reoperations (13.3%)
Xie et al. [24] (Group A)	Dislocation: 0%	Patellar instability without re-dislocation (2.4%)
Xie et al. [24] (Group B)	Dislocation: 4.7%	Patellar instability without re-dislocation (18.6%)
Zhang et al. [25]	Dislocation: 0%	Palpable pain in medial patella (8.3%), palpable pain in medial epicondyle (13.3%)

Discussion

The most significant findings from the present study include the following: 1) ROM and radiographic measurements improved regardless of surgical technique or presence of concomitant procedure; 2) the majority of studies in which pre- and postoperative PROs were reported demonstrated statistically significant improvements; and 3) the overall re-dislocation rate was 5.75%.

While there was a significant amount of heterogeneity with respect to the radiographic measurements and descriptions of range of motion, all studies that reported pre- and postoperative ROM showed significant improvement at the latest follow-up. The majority of studies found significant improvement, defined by PRO measures, at the latest follow-up for patients undergoing MPFL reconstruction, with and without the presence of concomitant procedures. Ten studies investigated the mid- to long-term outcomes of isolated MPFL reconstruction, one study compared isolated reconstruction with several concomitant procedures, and one study analyzed outcomes of MPFL reconstruction with one concomitant procedure. Marcheggiani Muciolli et al. utilized a fascia lata allograft with and without patellar stabilizing surgeries, including distal tibial tuberosity (TTA) distalization, TTA medialization, and TTA distalization/medialization with trochleoplasty. The group found that, regardless of the presence of concomitant procedures, 94% of patients saw improvement in knee function, while 50% returned to the pre-injury level of competitive sports participation [20]. Shimizu et al. utilized a semitendinosus autograft and performed an MPFL reconstruction with Insall’s proximal realignment. The study found similar improvements in PROs compared to those without additional procedures [23]. The design and results of the study by Xie et al. were unique in several respects. While all subjects underwent MPFL reconstruction with semitendinosus tendon grafts, patients were divided into two groups: semitendinosus tendons with or without polyester suture augmentation. Intriguingly, the study found a significant difference in failure rates, defined by recurrent patellar instability or re-dislocation, with those undergoing polyester suture augmentation demonstrating much lower failure rates [24].

These results indicate the need for future analysis regarding the use of suture augmentation not only in MPFL reconstruction procedures, but in other ligament repairs and reconstructions as well. A group in Australia are currently in the process of performing a randomized trial with the use of suture tape augmentation for single bundle anterior cruciate ligament (ACL) reconstructions. They hypothesize that patients will demonstrate decreased residual knee laxity and increased PROs at a two-year follow-up [26].

Gracilis tendon autografts were the most common sources for ligament reconstruction with semitendinosus grafts being the second most commonly used. Hamstring tendon grafts are extremely versatile and used for several types of ligament reconstruction including ACL and coracoclavicular ligament procedures [27]. The findings in the present study indicate hamstring tendon use is preferable for long-term stability and low re-dislocation rates. A study by Karnovsky et al. found that gracilis and semitendinosus graft use in tibialis anterior reconstruction resulted in similarly favorable outcomes. At a minimum six-month follow-up, patients were found to have restored ankle range of motion, significantly improved tendon strength on inversion and dorsiflexion, and few deficits comparing affected and unaffected ankles [28]. Nomura et al. investigated the use of polyester tape for MPFL reconstruction, which demonstrated significant improvement in Kujala scores and two instances of re-dislocation, a comparable number with the remaining studies [21]. A 2016 study by Wagih et al. described the use of polyester tape for anterolateral knee ligament (ALL) reconstruction and found it to be a feasible option for ALL procedures [29]. Given the results of the present study, hamstring autografts appear to be the most favorable option during isolated MPFL reconstruction or MPFL reconstruction with concomitant procedures. However, further investigation is warranted to determine the most ideal graft source.

The findings in the present study are consistent with existing literature and further the stance regarding the positive outcomes of MPFL reconstruction for recurrent patellar instability. A systematic review conducted in 2016 regarding short to mid-term outcomes of MPFL reconstruction found that, at the latest follow-up, a high percentage of patients returned to sports and that short-term results demonstrated a low incidence of postoperative apprehension or recurrent instability and low reoperation rates [30]. However, mid- to long-term outcomes cannot be viewed as a consequence of the efficacy of the procedure alone, but are heavily impacted by rehabilitation protocols and adherence as well. A 2017 study by Manske et al. described in meticulous detail the most ideal rehabilitation protocol for patients following MPFL reconstruction consisting of four phases lasting up to 21 weeks. Range of motion is gradually increased with the goal of protecting the newly reconstructed ligament, while also decreasing muscle atrophy due to lack of use [31]. Interestingly, there was variation in rehabilitation protocols from study to study. Gupta et al. placed patients in a brace fully immobilized for two weeks [17], while Marcheggiani Muccioli et al. encouraged patients to begin isometric exercises from postoperative day one [20]. While both studies demonstrated improved PROs, the former reported four cases of positive apprehension, while the latter did not. Future investigation is warranted regarding optimal rehabilitation and exactly how the protocols and adherence impact long-term outcomes.

Limitations

The findings of the present study must be considered through the context of its limitations. First, there was a tremendous amount of heterogeneity among the included studies with regard to graft source, surgical technique used, presence of concomitant procedures, inclusion or exclusion of trochlear dysplasia patients, and presence of pre-operative cartilage lesions. Hence, the overall outcomes presented may be impacted. Second, measures of clinical benefit, a minimal clinically important difference (MCID) and patient-acceptable symptomatic state (PASS), were not reported, and while PROs are beneficial in assessing outcomes, clinical benefit measures assist surgeons in determining true postoperative improvement. Third, radiographic measurements and definitions of range of motion varied heavily, making final determinations regarding these metrics difficult. Fourth, revision and failure rates were not widely reported; hence, conclusions regarding these rates cannot be determined from the present study. Finally, though a comprehensive database search was performed, some studies could have potentially been missed and not included in this review.

## Conclusions

The studies included in the present systematic review and meta-analysis demonstrate that MPFL reconstruction results in excellent PROs with low re-dislocation rates at a minimum five-year follow-up. While the procedure has been shown to be safe and efficacious in the short term, the findings of the present study affirm that it can be safely considered in patients with patellar injuries or patellar instability in the long term.
